# Cesarean delivery on maternal request and Robson classification

**DOI:** 10.1002/ijgo.70841

**Published:** 2026-02-02

**Authors:** Cristine Moreira Silva Benetti, Célia José Laice Sitoe Muhandule, Laura Bianchini Fogulin, Helymar Da Costa Machado, Eliana Amaral

**Affiliations:** ^1^ School of Medical Sciences University of Campinas (Unicamp) Campinas São Paulo Brazil; ^2^ Statistics Department Prof. Dr. José Aristodemo Pinotti Women's Hospital (CAISM/Unicamp) Campinas São Paulo Brazil

**Keywords:** cesarean section, choice behavior, high‐risk pregnancy, induced labor, obstetrics

## Abstract

**Objective:**

To describe the types of cesarean delivery on maternal request (CDMR), and to analyze the distribution of delivering types according to the Robson Classification (RC) and the distribution of comorbidities among the different groups of delivering types.

**Methods:**

Cross‐sectional study using electronic records of deliveries that occurred in a public, academic high‐risk maternity hospital in Brazil, from October 2017 to October 2021. CDMR included first or second cesarean delivery (CD), subdived into three groups: elective (CDMRele), induction withdrawal (CDMRiw), and labor withdrawal (CDMRlw).

**Results:**

In 7779 deliveries, 53.1% (4132) were CD. The CDMR group corresponded to 10.2% (420/4132) of all CD and 5.4% (420/7779) of all deliveries. CDMRele were the main group (246 – 58.6%), followed by CDMRiw (118 – 28.1%), and CDMRlw (56 – 13.3%). The CDMR and its subdivisions were concentrated in RC group 5.1 (CDMR 240 – 57.1%, CDMRele 156 – 63.4%, CDMRiw 54 – 45.7% and CDMRlw 30 – 53.6%). Second CD corresponded to 3.1% (247/7779) of all deliveries and 58.8% (247/420) of CDRM. Among CDMR, 72.1% (303/420) of patients had comorbidities and the most frequent diseases were hypertension and diabetes.

**Conclusion:**

The definition of CDRM in the international literature is not a consensus and Brazil's law supports CDMR in circumstances not previously described. The RC is limited for understanding the growing phenomenon of CDMR among a group of pregnant women with high prevalence of comorbidities.

## 1 INTRODUCTION

Cesarean delivery (CD) is the most common surgery in the world, with different rates in different countries and regions.^1^ Places with a high Human Development Index have high rates of CD, whereas places with a low Human Development Index have lower rates. Nevertheless, these differences do not reflect the usual distribution of clinical risk conditions that could require more CD. Some countries, due to health inequality, face both problems, depending on social, economic, and cultural practices.^1^, ^2^

CD increases infection rates, postpartum hemorrhage, blood transfusion, organ damage, and anesthetic complications, as well as placenta accreta and uterine rupture in future pregnancies.^3^ There are also potential negative perinatal outcomes, including prematurity, neonatal and postnatal asphyxia, and increasing risk for later diseases such as diabetes, obesity, asthma, autoimmune diseases, autism spectrum disorders, hyperactivity, and attention deficit.^4^, ^5^ Considering the potential complications impacting women's and infant's health, unjustified CD must be avoided.

The World Health Organization recommends the Robson Classification (RC) as standard to study and compare data between different locations. It is based on five parameters (parity, number of fetuses, gestational age, fetal presentation, and onset of labor) that classify pregnant woman in 10 different groups, which are totally inclusive and mutually exclusive.^6^

Cesarean delivery on maternal request (CDMR), a recent phenomenon, has been contributing to the increased rates of CD. The first articles on the issue, published in the late 1980s, raised the ethical debate around accepting the woman's wish for a CD without a clinical reason ^7^ Nevertheless, the CDMR definition is controversial.^8^ The more commonly used definition states that CDMR is a “primary CD requested by woman, without any maternal or fetal medical indication that justifies the procedure”.^9^ On the other hand, the National Institute for Health and Care Excellence (NICE) and the Royal Australian and New Zealand College of Obstetricians and Gynecologists (RANZCOG) do not restrict the concept and accept all CD requested by a woman, without medical indication.^10^, ^11^

Brazil became a leader in CD rates and recent laws offer pregnant woman the right to choose a CD after 39 weeks of pregnancy. The perception of CD as a predominant mode of delivery, the labor seen as an experience involving extreme suffering, and social intolerance for unexpected negative perinatal outcomes, especially during vaginal delivery (VD), add layers to the decision making process for women and their families, facilitating the request for CD.^1^, ^12^, ^13^

## 2 MATERIALS AND METHODS

We performed a cross‐sectional study using electronic records of all deliveries taking place at a public university maternity service in southeast Brazil, from October 2017 to October 2021. This tertiary hospital receives pregnant woman from different cities and provides advanced medical care and treatment services for complex and severe conditions. The categories of deliveries included VD, CD on medical indication (CDMI), and CDMR. For CDMR, we included first and second CD and proposed a subdivision into three groups, including: (1) CDMR—elective (CDMRele): planned CD in patients that received an explanation about delivery modes and refused labor induction or waiting for spontaneous labor; (2) CDMR—induction withdrawal (CDMRiw): patients that agreed with and started labor induction, but gave up during the process; and (3) CDMR—spontaneous labor withdrawal (CDMRlw): patients in spontaneous labor that asked for CDMR.

The frequency and percentage distribution of delivery types according to the RC modified^6^ and maternal comorbidities were tabled and groups were compared using the *χ* ^2^‐test. The most common diseases were listed according to frequency and percentage.

This study was approved by the Research Ethics Committee (# 65705922.0.0000.5404), was exempted from using the Free and Informed Consent Form, and adopted the Data Use Commitment Form.

## 3 RESULTS

**FIGURE 1:**
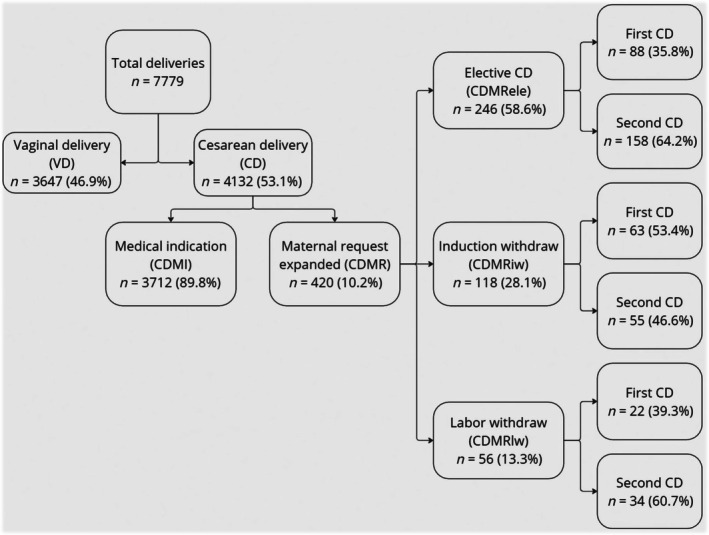
Route of delivery at a Brazilian reference maternity service.

Figure 1 shows the distribution of deliveries during the study period. Among 7779 registries, 53.1% (4132) were CD, divided between 89.8% (3712) CDMI and 10.2% (420) CDMR. CDMR, totaling 5.4% (420/7779) of all deliveries, were subdivided into elective (246 – 58.6%), induction withdrawal (118 – 28.1%), and labor withdrawal (56 – 13.3%). Among CDMRele, 64.2% (158) were a second CD, as well as 46.6% (55) of CDMRiw and 60.7% (34) of CDMRlw (Figure 1).

Table 1 shows the distribution of comorbidities among types of delivery groups. Most patients with VD (2714/3647 – 74.4%), CDMRlw group (35/56 – 62.5%) and CDMI (2150/3712 – 57.9%) had no disease, but 72.1% (303/420) of the pregnant women in the CDMR group had comorbidities.

**TABLE 1:** Distribution of comorbidities among types of delivery groups.^a^

Comorbidities	CDMRele	CDMRiw	CDMRlw	CDMR	CDMI	VD	Total
Yes	184 (74.8)^b^	98 (83.0)^b^	21 (37.5)^b^	303 (72.1)^b^	1562 (42.1)^b^	933 (25.6)	2798 (36.0)
No	62 (25.2)	20 (17.0)	35 (62.5)	117 (27.8)	2150 (57.9)	2714 (74.4)	4981 (64.0)
Total	246 (3.2)	118 (1.5)	56 (0.7)	420 (5.4)	3712 (47.7)	3647 (46.9)	7779

Abbreviations: CDMI, cesarean delivery on medical indication; maternal request; CDMR, cesarean delivery on maternal request; CDMRele, CDMR elective; CDMRiw, CDMR—induction withdrawal; CDMRlw, CDMR—spontaneous labor withdrawal; VD, vaginal delivery.

^a^ Data are presented as number (percentage).

^b^ *χ* ^2^ test: *P* = 0.043 (VDXCDMRlw); *P* < 0.001 (VDXCDMI; VDXCDMR; VDXCDMRiw; VDXCDMRele).

Table 2 describes the distribution of comorbidities between delivering types. Hypertension and diabetes were the most frequent diseases in general, but also among the CDMI group.

**TABLE 2:** Most frequent comorbidities among route of delivery groups.^a^

Comorbidities	CDMRele	CDMRiw	CDMRlw	CDMI	VD	Total
Hypertension	37	26	3	553	208	827
Diabetes	42	25	6	433	281	787
Other diseases	25	8	8	292	186	519
Endocrinopathy	20	6	2	199	160	387
Sexually transmitted infection	10	3	1	116	74	204
Anemia	7	2	3	84	42	138
Infection	6	–	–	58	32	96
Depression	2	0	1	51	40	94
Heart disease	3	0	0	53	23	79
Pregnancy complications	3	2	–	41	13	59
Epilepsy	3	2	1	34	15	55
Collagenosis	4	3	–	30	17	54
Thromboembolism	2	–	–	32	19	53
Infectious disease	–	–	–	18	23	41
Previous surgery/hospitalization	2	–	–	25	8	35
Nephropathy	1	1	0	21	10	33
Victim of violence	2	0	0	4	0	6

Abbreviations: CDMI, cesarean delivery on medical indication; maternal request; CDMR, cesarean delivery on maternal request; CDMRele, CDMR elective; CDMRiw, CDMR—induction withdrawal; CDMRlw, CDMR—spontaneous labor withdrawal; VD, vaginal delivery.

^a^ Data are presented as number.

Table 3 shows the types of delivery distribution according to RC, with 5483 (70.5%) deliveries from groups 1 to 5.1. Among these, 60.3% (3305) were in primiparous women with cephalic presentation at term (groups 1 to 2B). VD was concentrated among women in spontaneous labor, cephalic presentation, term (groups 1 and 3). CDMI was mostly found in groups 2B (primiparous women, cephalic presentation at term with induced labor) and 10 (cephalic presentation, preterm, including previous CD).

**TABLE 3:** Distribution of types of delivery by Robson groups.^a^

Robson groups	CDMRele	CDMRiw	CDMRlw	CDMR	CDMI	VD	Total
1	–	–	6 (10.71)	6 (1.42)	349 (9.40)	1236 (33.89)	1591 (20.45)
2A	–	47 (39.83)^b^	–	47 (11.19)	283 (7.62)	589 (16.15)	919 (11.81)
2B	56 (22.76)^b^	–	10 (17.85)^b^	66 (15.71)^b^	729 (19.63)^b^	–	795 (10.21)
3	–	–	3 (5.35)	3 (0.71)	95 (2.55)	686 (18.80)	784 (10.07)
4A	–	12 (10.16)	–	12 (2.85)	117 (3.15)	402 (11.02)	531 (6.82)
4B	25 (10.16)^b^	–	2 (3.57)^b^	27 (6.42)^b^	310 (8.35)^b^	–	337 (4.33)
5.1	156 (63.41)^b^	54 (45.76)^b^	30 (53.57)^b^	240 (57.14)^b^	181 (4.87)^b^	105 (2.87)	526 (6.76)
5.2	–	–	–	–	266 (7.16)^b^	–	266 (3.41)
6	–	–	–	–	266 (7.16)^b^	44 (1.20)	310 (3.98)
7	–	–	–	–	192 (5.17)^b^	41 (1.12)	233 (2.99)
8	3 (1.21)	–	1 (1.78)^b^	4 (0.95)	195 (5.25)^b^	48 (1.31)	247 (3.17)
9	–	–	–	–	44 (1.18)^b^	–	44 (0.56)
10	6 (2.43)^b^	5 (4.23)	4 (7.14)	15 (3.57)	685 (18.45)^b^	496 (13.60)	1196 (15.37)
Total	246 (3.2)	118 (1.5)	56 (0.7)	420 (5.39)	3712 (47.7)	3647 (46.9)	7779

Abbreviations: CDMI, cesarean delivery on medical indication; maternal request; CDMR, cesarean delivery on maternal request; CDMRele, CDMR elective; CDMRiw, CDMR—induction withdrawal; CDMRlw, CDMR—spontaneous labor withdrawal; VD, vaginal delivery.

^a^ Data are presented as number (percentage).

^b^ *χ* ^2^‐test: *P* < 0.001 (VDXCDMI; VDXCDMR; VDXCDMRlw; VDXCDMRiw; DXCDMRele).

Most CDMR were observed for group 5.1 (term, cephalic, previous CD). CDMRele was the most frequent category of CDMR, more requested by nulliparous or multiparous women with previous CD, cephalic presentation, at term (groups 2B and 5.1). CDMRiw was mostly observed among induced primiparous and multiparous women with a previous CD (groups 2A and 5.1). CDMRlw were frequent between woman with a previous CD.

## 4 DISCUSSION

Our findings showed that 5.4% (420/7779) of all deliveries and 10.2% (420/4132) of all CD were CDMR, whereas elective CD corresponded to 3.2% (246/7779) of all deliveries and 58.6% (246/420) of all CDMR. A global meta‐analysis showed that 3% of all deliveries are elective CDMR, and that 11% of the total number of world CD are CDMR. Ireland was the country with the lowest rate of CDMR (0.9%) and China is the country with the highest rate (60%).^4^ So, our findings are similar to those observed elsewhere.

Primary elective CDMR corresponded to 1.1% (88/7779) of all deliveries and 20.9% (88/420) of CDMR, fitting the American College of Obstetricians and Gynecologists (ACOG) CDMR definition.^9^ Nevertheless, multiparous woman with a previous CD also request CD (247/7779 – 3.2% of all deliveries and 247/420 – 58.8% of CDMR), this group fits the NICE and RANZCOG definition.^10^, ^11^

We did not find in the literature the situation of a woman that agrees with labor induction and then gives up during the process (CDMRiw: 118/7779 – 1.5% of all deliveries and 118/420 – 28.1% of CDMR), and woman in whom the labor process starts spontaneously, but they do not want to continue and ask for CD (CDMRlw: 56/7779 – 0.7% of all deliveries and 56/420 – 13.3% of CDMR). Maternal desire for CD has been studied in two ways in the literature: (1) the woman's preference for CD during prenatal care, and before labor starts or (2) after delivery, when the CDMR has already been performed. We were unable to find any study focusing on the conditions that affect women's choices and decisions among a high‐risk population, even without a medical indication for CD, supported by laws and a culture of CD as the natural mode of delivering, as observed in Brazil.^14^, ^15^, ^16^

This maternity hospital is located in São Paulo state, Brazil, which allows pregnant women to opt for a CD, which can be performed from 39 weeks of pregnancy without medical indication.^13^ This legal framework helps to create a more permissive environment to any request from women, observed even after induction or labor starts. Brazil has had high rates of CD for many decades, so many doctors and health professionals were trained predominantly on CD, whereas women have not experienced VD, neither their families nor their friends. These conditions render delivering by CD almost natural. In addition, the judicialization of health corroborates with this situation.^2^

Such an attitude towards CDMR is not without conflict among obstetricians. Obstetric guidelines have a common position about CDMR, considering the principle of patient autonomy. ACOG, NICE, RANZCOG, ISS (Italian Nacional Institute of Health), and SOCG (the Society of Obstetricians and Gynecologists of Canada) consider CDMR after appropriate information and counseling. On the other hand, respecting the other ethical principles (beneficence, non‐maleficence, and justice), the physician can agree or disagree with the maternal choice and refer the patient for a second opinion.^17^, ^18^

A qualitative study in the same population of this study, with women who requested CD shows that women do have fear of labor pain, feel themselves safer avoiding maternal/fetal increased risks, had themself/friend/family traumatic labor experience, felt more control about the situation, and lacked knowledge about the risks and benefits of CD.^19^

Based on this safety feeling of CD when compared with VD, we observed that CDRM were frequent (303/420 – 72.1%) between woman who also have a disease, even when there is no medical indication for CD.

The CDMRlw group might be motivated to ask for a CD because of labor pain.^20^ Among the CDMRele we found many cases of women that came to prenatal care in this referral service because of their disease and delivering had to be anticipated according to the protocols in use (for pre‐eclampsia or diabetes for example). Induced labor is offered as an alternative, considering our clinical staff have a large experience with VD and instrumented VD if necessary, but many patients do not consent, or consent and later change their opinion, resulting in a CDMR.^21^, ^22^

This study is not free of limitations. We used an electronic data bank of obstetric cases, and misclassification of reasons to request a CD may have occurred. We tried to reduce this bias by revising all CDMR. Also, the institution is a referral center for high‐risk pregnancies in a wealthy state and region, in a country with high rates of primary CD. Understanding the complex interaction between women, their families, and health professionals during prenatal care and labor is necessary to understand the reasons for their choice since the first delivery, as it occurs in populations that perceives CD as a natural mode of delivery.

It is well known that RC can provide a framework for systematic auditing of several obstetric conditions, such as postpartum hemorrhage, obstetric anal sphincter injury and Apgar score, or pre‐eclampsia.^23^, ^24^ Our project aimed to revise the contribution of the RC to improving understanding of the CDMR phenomenon. Nevertheless, as it does not consider the personal and clinical reasons that led to maternal decision making regarding mode of delivery, we can see a restricted role for RC in this regard when there is a complex interaction of factors including high‐risk pregnancies in a pro‐CD culture and legislation environment.

In conclusion, while the majority of CDMR are still elective deliveries, requesting a second CD, giving up spontaneous labor, and labor induction were also relevant associated conditions. The RC was a limited option to better understand the phenomenon of CDMR in a referral center for high‐risk pregnancies. In a country where women's demand on mode of delivery is built culturally with legal support, the definition of CDMR need to be expanded.

## AUTHOR CONTRIBUTIONS

CMSB, CJLSM, and LBF made substantial contributions to the conception of the work, with data acquisition and interpretation of data for the work; HDCM made substantial contributions to the conception of the work, with statistical analysis; and EA critically reviewed the work, gave final approval of the version to be published, and is accountable for all aspects of the work in ensuring that questions related to the accuracy or integrity of any part of the work are appropriately investigated and resolved.

## FUNDING INFORMATION

The authors declare that no financial support was received for the research, authorship, and/or publication of this article.

## CONFLICT OF INTEREST STATEMENT

The authors have no conflicts of interest.

## Data Availability

Data available on request due to privacy/ethical restrictions.
